# Romosozumab and cardiovascular safety in Japan

**DOI:** 10.1007/s00774-025-01596-w

**Published:** 2025-03-09

**Authors:** Yasuhiro Takeuchi

**Affiliations:** 1 Toranomon Hospital Endocrine Center, 2-2-2 Toranomon, Minato-ku, Tokyo, 105-8470 Japan; 2 Okinaka Memorial Institute for Medical Research, Tokyo, Japan

The Letter to the Editor from Dr. Kawaguchi provides an issue that needs to be more seriously addressed. Some real-world data suggest that romosozumab is associated with the incidence of cardiovascular (CV) events in patients with osteoporosis, although others do not support their association. His argument that the death rate, presumably the majority of them being associated with CV events, in Japan may be higher in patients with romosozumab than other osteoanabolic agents is primarily based on the JADER database of romosozumab compared with teriparatide [ref. 5 in Kawaguchi’s letter]. The JADER database is a spontaneous reporting system of drug adverse events which is managed by the Pharmaceuticals and Medical Devices Agency (PMDA) in Japan. More than 90% of reports have been submitted by professional healthcare providers, many of them through pharmaceutical companies. In contrast, Masuda S et al. have reported that the weighted incidence rate difference (IRD) for MACE (major adverse cardiovascular event) between romosozumab versus teriparatide was –0.06 (95% CI, –0.20 to 0.09) events per 100 person-years (weighted HR, 0.90 [95% CI, 0.68 to 1.19]) in Japan based on their investigation conducted an active comparator, a new user cohort design, with confounding controlled by inverse probability of treatment weighting using a commercial administrative claims database provided by DeSC Healthcare, Inc. (March 2019 to October 2022) [originally cited as ref. 46 in Takeuchi’s article]. Unlike outcomes based on spontaneous reporting like the JADER database, the study cited above used professionally documented ICD-10 codes from patient electronic medical records to define CV outcomes. In addition, electronic medical records were used to obtain extensive data regarding baseline CV risk, and cohorts were meticulously balanced accordingly using propensity score matching. Thus, Masuda S et al. may succeed in directly demonstrating the CV safety profile of romosozumab compared to PTH analogs.

Recently, PMDA issued on January 31, 2025, the results that the frequency of CV events after prescription of romosozumab in Japan was compared with that of teriparatide (recombinant) or teriparatide acetate to evaluate the association between romosozumab and CV events using the anonymous database of medical insurance-related information (NDB) [1]. The patients who were prescribed romosozumab or teriparatide from March 4, 2019 to March 31, 2023 were included. No major differences were observed in the patient backgrounds of the exposed and control groups overall, although differences were observed in the proportion of males and the proportion of patients who received a prescription for anticoagulant medication. The results of the evaluation of the association between romosozumab and the occurrence of CV events showed no consistent trend toward an increased risk with romosozumab compared with teriparatide, regardless of gender or history of CV events. Similar results were observed among cases, even though their CV events had been recorded within 1 year at the time of the first prescription of romosozumab. Patients with a history of CV events were included, 5.0% and 6.9% of those with romosozumab and teriparatide, respectively. In more detail, 0.6% and 1.2% had CV events within 1 year at the time of the first prescription of romosozumab and teriparatide, respectively.

For analyzing data reflecting the real-world clinical evidence in Japan, there is a reservation when investigations with the JADER database are evaluated. It has been mandatory for pharmaceutical companies in Japan to submit a risk management plan (RMP) as part of the approval process for new drugs since 2013. Romosozumab was launched after 2013 but teriparatide (recombinant) and teriparatide acetate (once-weekly) were launched before 2013. As the RMP of romosozumab, they define CV events as potential risks that need to be managed by healthcare providers and pharmaceutical companies. This fact may be associated with differences in the spontaneous (voluntary) reported incidence of CV events and death related to CV events in patients with romosozumab from teriparatide. As Dr. Kawaguchi has claimed, we should reduce the risk of CV events in osteoporotic patients with a higher risk of fracture by observing a warning and precaution of the drug package insert because there remains a concern about increased CV events in those patients.
